# Mastopexy for secretory carcinoma of breast in a young female: a case report

**DOI:** 10.1093/jscr/rjae351

**Published:** 2024-06-11

**Authors:** Ghazanfar Ali, Kumail Ijaz, Mishal Ihsan, Muhammad Usama, Syed Muhammad Shabbir Ali Naqvi

**Affiliations:** Department of Surgery, Mayo Hospital Lahore, Lahore 54000, Pakistan; Department of Surgery, Mayo Hospital Lahore, Lahore 54000, Pakistan; Department of Surgery, Mayo Hospital Lahore, Lahore 54000, Pakistan; Department of Surgery, Mayo Hospital Lahore, Lahore 54000, Pakistan; Khyber Medical University Peshawar, Institute of Public Health and Social Science IPH&SS Peshawar 25000, Pakistan

**Keywords:** secretory carcinoma, vertical mastopexy, breast surgery, case report

## Abstract

Secretory carcinoma, a triple-negative benign tumor, is one of the rarest malignancies of the breast which rarely metastasizes. Surgical excision via lumpectomy or mastectomy is the mainstay of treatment, but in young patients, mastopexy can be a better option cosmetically. A 26-year-old woman presented with a lump in her right breast that, on ultrasonography, was revealed to be a multi lobulated solid lesion measuring 25 × 16 mm^2^ in the retro areolar region at a 4 o’clock position. It turned out to be secretory carcinoma of the breast in a tru-cut biopsy. Vertical Mastopexy was opted for the removal and simultaneous reconstruction of the breast, which was followed by adjuvant chemotherapy and radiotherapy. Vertical mastopexy showed that the tumor was removed, and the breast was restored to its original form simultaneously. This procedure gave better results clinically and cosmetically. The patient had an uneventful recovery and is on a regular follow-up.

## Introduction

Secretory carcinoma is one of the rare malignancies of the breast. Only ≤0.05% of the cases of invasive breast carcinomas belong to this histological subtype [1]. It was initially described as ‘Juvenile Breast Carcinoma’ by McDivitt and Stewart in 1966 [2]. But now it is known to prevail in pediatric age groups as well as adolescent women [3]. Histologically, it is a triple-negative tumor that comprises of low-grade nuclei and nests of intracellular and extracellular secretory material separated by collagenous bands [4]. This tumor shows benign characteristics and rarely metastasizes. Surgical excision via lumpectomy or mastectomy is the mainstay of treatment but a mastopexy technique can also be used to improve the cosmetic appearance of breast [5]. As it is a rarest subtype of breast carcinoma, therefore, there are very few cases of secretory carcinoma of breast reported in Pakistan. Most of the cases of breast carcinomas have been seen in the women above the age of 40 This article presents a case of secretory carcinoma of breast in a very young female with favorable prognosis. Vertical mastopexy was performed instead of mastectomy because it provides better cosmetic appearance. It was followed by chemotherapy. Patient is doing quite well now.

## Case report/presentation

A 26-year-old woman presented with a lump in her right breast that was detected by the patient on self-examination. The patient had no past medical history or family history of breast cancer.

**Figure 1:**
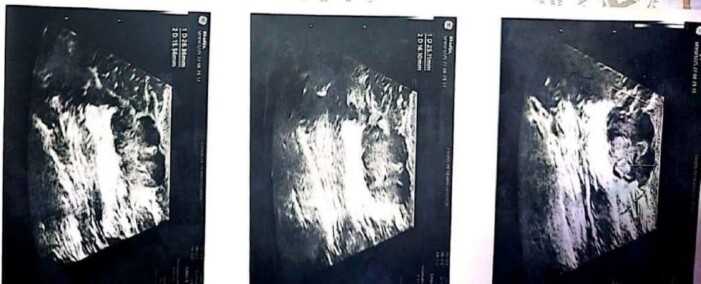
Breast ultrasonography.

**Figure 2:**
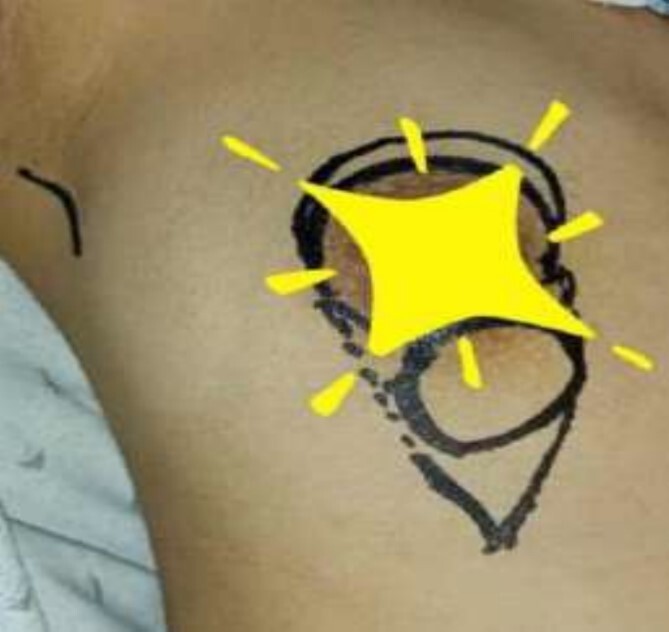
Mastectomy procedure (incision outline).

During a physical examination, the patient had a well-circumscribed palpable mass of 2 cm below the nipple at a 6 o’ clock position (Fig. 2). She had no associated pain, nipple retraction or any skin changes. Examination of the axilla and left breast was unremarkable. Axillary and internal mammary lymph nodes were not palpable. Breast ultrasonography revealed a multi lobulated solid lesion measuring 25 × 16 mm^2^ in the retro areolar region at a 4 o’clock position on the right side just beneath the nipple (Fig. 1b). Left breast showed normal parenchyma. There was no calcification and skin thickening. Lymph node status was negative on imaging.

Then a Tru-cut biopsy was done which revealed secretory carcinoma. This section showed a neoplasm made up of solid nests of tumor cells as well as glands. The tumor cells had abundant amphophilic cytoplasm and mild atypia. There was secretion of eosinophilic material within the cribriform spaces. Immunohistochemical staining showed the following; SOX10 positive, S100 positive, p63 negative, ER negative.

**Figure 3:**
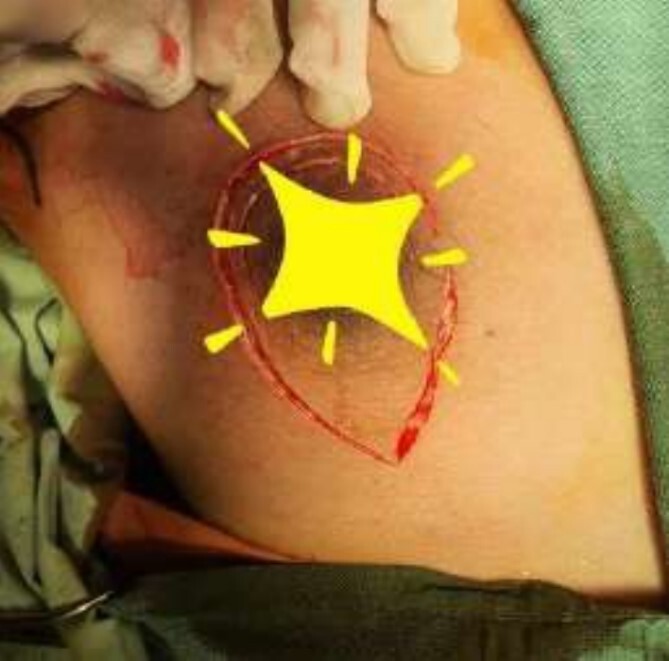
Mastectomy procedure (Elliptical Incision given around nipple).

**Figure 4:**
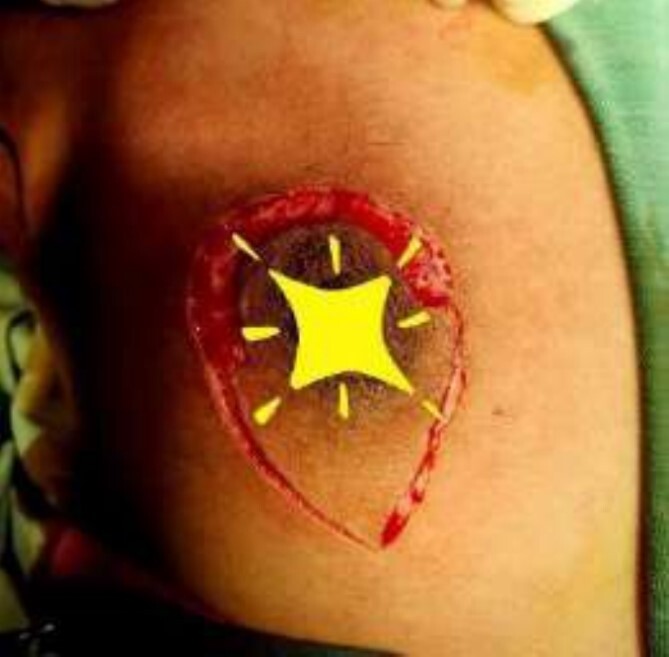
Mastectomy procedure (Nipple Flap Created).

**Figure 5:**
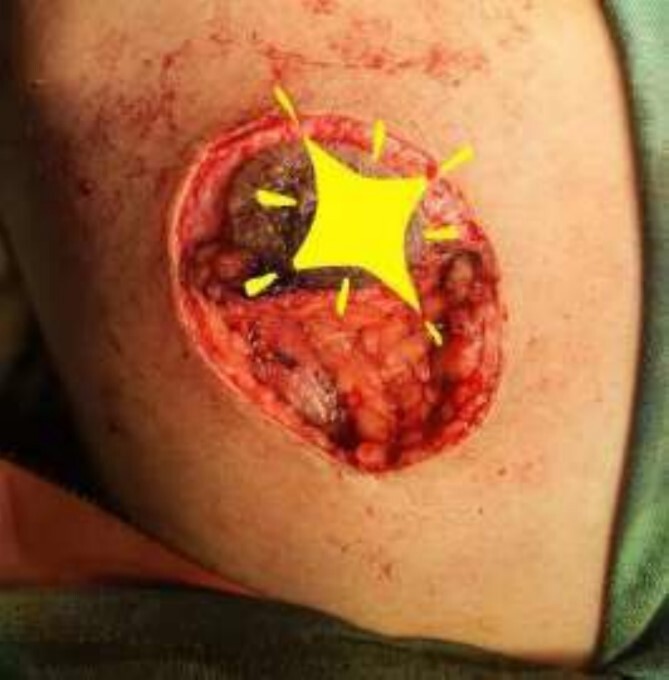
Mastectomy procedure (Nipple Flap Removal).

**Figure 6:**
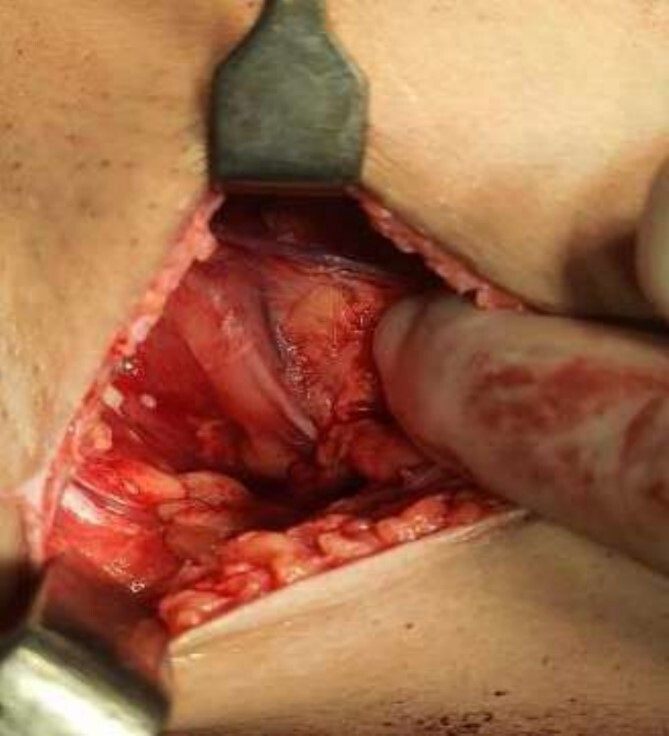
Mastectomy along with sentinel lymph node biopsy procedure (Tumor Approach).

**Figure 7:**
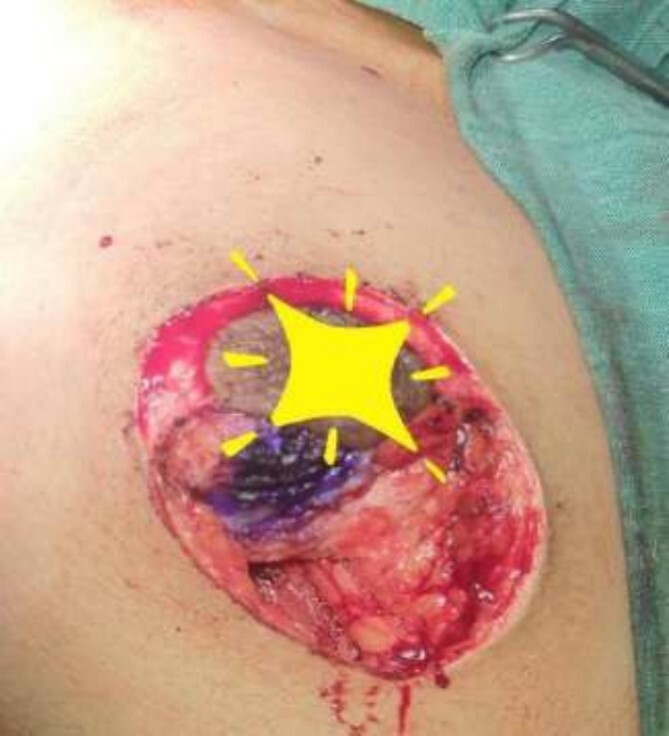
Mastectomy along with sentinel lymph node biopsy procedure (Sentinel Lymph node Biopsy).

The operating surgeon opted for a vertical mastopexy to remove the mass and simultaneously reconstruct the breast to restore it as much as possible and to have a cosmetically better scar (Fig. 7). The skin was marked, a peri-areolar ellipse having a diameter of about 15 cm was made that extended vertically below, 4 cm above the inframammary fold. The skin was dissected along the incision line and the mass was removed. Grossly, the specimen measuring 5 × 3.5 × 3 cm^3^, containing the firm mass, was sent for histopathological analysis. Axillary lymph node biopsy was done. Two drains were placed, and the skin was closed in the form of a superior circle and an inferior ellipse (Figs 3–6).

**Figure 8:**
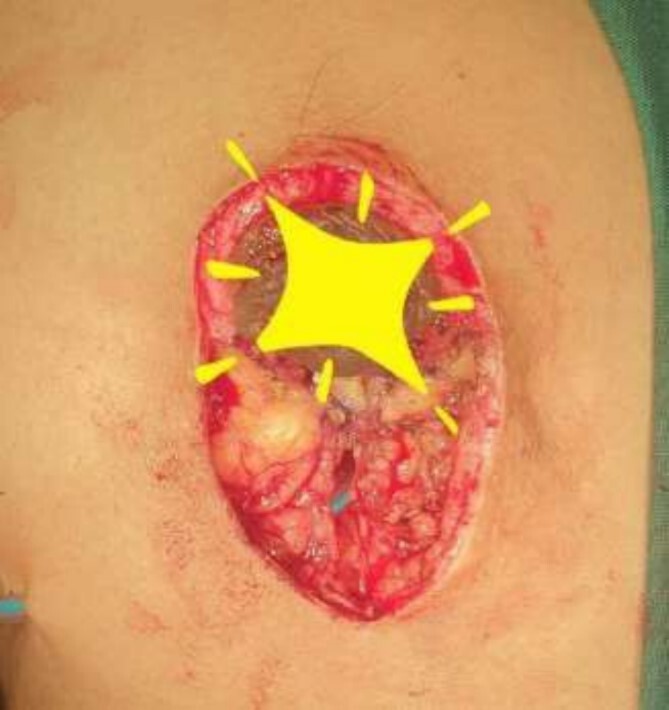
Mastectomy along with sentinel lymph node biopsy procedure (Tumor extracted).

The frozen section diagnosis revealed secretory carcinoma, unifocal, 2.3 × 1.7 × 1.5 cm^3^ in size. Skin was unremarkable and free of tumor. There was no intraductal component. All the resection margins were free of tumor and the closest superior margin was free by less than 0.1 cm. A total of 16 lymph nodes were isolated from axillary tissue, all of which were negative for metastatic carcinoma (0/16). No venous, lymphatic or perineural invasion was identified. Pathological staging revealed primary tumor; pT2, lymph nodes; pN0, distant metastasis; pMx. Estrogen and progesterone receptors were negative (Fig. 8).

**Figure 9:**
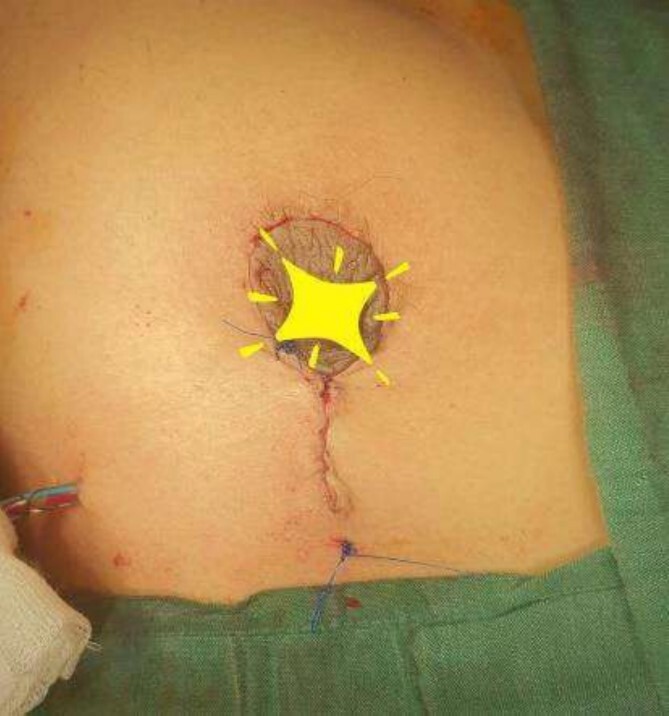
Vertical mastopexy.

**Figure 10:**
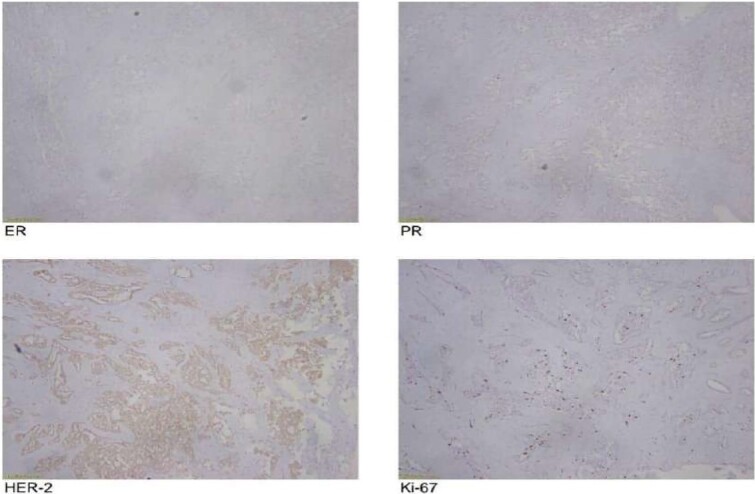
Pathological staging of tumor.

She was advised adjuvant chemotherapy and radiotherapy. It consisted of three cycles of adriamycin and cyclophosphamide regimen followed by 3 cycles of taxane. She is tolerating it well and is on a regular follow-up. She has been disease-free since then.

## Discussion

Secretory carcinoma of breast is one of the invasive diseases of the breast. It usually follows a benign course but in rare conditions it can metastasize to distant tissues. It was originally described as cancer of pediatric age group but later on it was found to occur in adolescent women as well. Prados *et al.* presented a case of 32-year-old woman with multicentric SCB but with a favorable prognosis [6]. Wang *et al.* described a case of 12-year-old girl who presented with a painless left-sided breast lump and underwent the sentinel lymph node biopsy and breast conserving therapy without any adequate benefits from chemotherapy [7]. Tang *et al.* (2021) reported a case of a 39-year-old woman with left sided SBC resistant to neoadjuvant chemotherapy and unfavorable prognosis [8]. Though the patient was treated by total mastectomy and axillary dissection, she developed multiple distant metastases in brain and died 19 months after the diagnosis. Previous literature states the cases of Sscretory carcinoma of breast either in old-aged women or pediatric population, while this case reports a case in a young 26-year-old female.

On physical examination, it mimics fibroadenoma. On mammography, it appears as a distinct mass containing discrete nodular densities with irregular margins [9]. On ultrasonography, SBC appears as a round, well-circumscribed, single or multilobulated lesion with hypoechoic or hyperechoic internal features [10]. There can be associated skin thickening and calcification. In young females, ultrasonography is superior to mammography owing to the density of the breast tissue; hence, the former modality was preferred. Imaging alone doesn’t provide adequate diagnosis as SBC appears as a benign lesion or a well-differentiated malignant mass such as papillary, medullary and mucinous subtypes [9]. Diagnosis can only be confirmed on the basis of pathological testing and for that purpose biopsy must be done if lesion gives suspicious features on BI-RADS. Initially, due to the nature of the mass being very similar to a fibroadenoma, it was suspected to be of the same category but later, on a tru-cut biopsy, it came as the rarest subtype; secretory carcinoma of the breast.

On histological examination, tumor may contain the solid nests of tumor cells as well as glands. The tumor cells show atypia and eosinophilic or amphophilic vacuolated cytoplasm. Secretion of eosinophilic material within the cribriform spaces can be present both intracellularly and extracellularly [11]. SBC usually presents as triple-negative tumor on immunohistochemical staining. In this case, it was SOX10 positive, S100 positive, p63 negative, ER negative. Immunohistochemical staining and histological examination are necessary to differentiate this specific tumor from other types of breast malignancies.

The treatment for secretory carcinoma of breast is just like other subtypes. Complete surgical excision of tumor along with axillary dissection if necessary is usually carried out. Sentinel lymph node biopsy must be done as large tumors (> 2 cm) can metastasize to axillary lymph nodes. In such cases, axillary lymph nodes dissection is necessary.

It is very important to keep in view the social dilemmas and psychological impact of a breast carcinoma in a female patient and that too in females of younger age groups, the psychosocial effect can be manifold. Hence, a certain surgical technique had to be adopted to fully eliminate the tumor, provide a cosmetically better scar and reconstruct the breast in the same setting. Lumpectomy along with Mastopexy is a more suitable approach as it allows the surgeon to remove the tumor as well as maintain the cosmetic appearance of breast at the same time [12]. Vertical mastopexy, as done in this case, has provided better cosmetic outcome and can be considered a better option when operating on unmarried female patients. Adjuvant chemotherapy plays a minor role as treatment modality but adjuvant radiotherapy prevents the local recurrence of the disease after breast conservation surgery [13]. Local recurrence of tumor is common 20 years after surgery [14]; thus, a 20-year follow-up is necessary.

## Conclusion

SBC, a rare breast condition, manifests as a solid lesion with benign imaging features and minimal metastatic potential. Its diagnosis requires biopsy, histological and immunohistochemical analysis to differentiate it from other breast cancers. Typically, for triple-negative tumors, chemotherapy offers limited benefits. Excision via lumpectomy with a simultaneous vertical mastopexy approach enhances cosmetic outcomes and can be used as a preferable method especially for unmarried females. Post-operative radiotherapy can reduce local recurrence risk but necessitates a 20-year follow-up for reassessment.
